# Germline Progenitors Escape the Widespread Phenomenon of Homolog Pairing during *Drosophila* Development

**DOI:** 10.1371/journal.pgen.1004013

**Published:** 2013-12-19

**Authors:** Eric F. Joyce, Nicholas Apostolopoulos, Brian J. Beliveau, C. -ting Wu

**Affiliations:** Department of Genetics, Harvard Medical School, Boston, Massachusetts, United States of America; Stowers Institute for Medical Research, United States of America

## Abstract

Homolog pairing, which plays a critical role in meiosis, poses a potential risk if it occurs in inappropriate tissues or between nonallelic sites, as it can lead to changes in gene expression, chromosome entanglements, and loss-of-heterozygosity due to mitotic recombination. This is particularly true in Drosophila, which supports organismal-wide pairing throughout development. Discovered over a century ago, such extensive pairing has led to the perception that germline pairing in the adult gonad is an extension of the pairing established during embryogenesis and, therefore, differs from the mechanism utilized in most species to initiate pairing specifically in the germline. Here, we show that, contrary to long-standing assumptions, Drosophila meiotic pairing in the gonad is not an extension of pairing established during embryogenesis. Instead, we find that homologous chromosomes are unpaired in primordial germ cells from the moment the germline can be distinguished from the soma in the embryo and remain unpaired even in the germline stem cells of the adult gonad. We further establish that pairing originates immediately after the stem cell stage. This pairing occurs well before the initiation of meiosis and, strikingly, continues through the several mitotic divisions preceding meiosis. These discoveries indicate that the spatial organization of the Drosophila genome differs between the germline and the soma from the earliest moments of development and thus argue that homolog pairing in the germline is an active process as versus a passive continuation of pairing established during embryogenesis.

## Author Summary

Meiosis is a specialized cell division that permits the transmission of genetic material to following generations. A pivotal step for this process is the pairing and recombination between homologous chromosomes. In the case of Drosophila, which supports organismal-wide homolog pairing throughout development, it has been widely assumed that the homolog alignment occurring during meiosis in the adult gonad is an extension of the pairing established during embryogenesis. Here, we show that, contrary to this model, homologous chromosomes are unpaired in germline progenitors from embryogenesis to adulthood. This discovery refutes the presumption that homologous chromosomes are paired in Drosophila in all cell types and demonstrates that a specific form of chromosome organization, namely, homolog pairing, is a signature feature that distinguishes cells destined to be the soma from cells destined to be the germline.

## Introduction

During meiosis, the germline nucleus undergoes extensive reorganization to accurately align homologous chromosomes along their entire length, enabling them to recombine and ultimately segregate from one another. Outside of the germline, however, homolog pairing, if it occurs at all, is usually transient and localized to a particular chromosomal region [1]–[7]. Indeed, the individual somatic chromosomes of many eukaryotes occupy distinct territories in the nucleus [8]–[10], which would be expected to minimize interactions between homologous chromosomes and thus pairing-mediated changes in gene expression, chromosome entanglements, and loss-of-heterozygosity due to mitotic recombination [11]–[17]. Consequently, extensive homolog pairing is generally considered a germline-specific phenomenon that is restricted to the early stages of meiosis.

One striking exception is found in Dipteran insects, such as Drosophila, where there is widespread homolog pairing in somatic cells. Such pairing has been documented in embryonic, larval, and adult tissues, with pairing frequencies at individual loci reaching 80% or more [18]–[23]. These observations have led researchers to speculate that Drosophila represents a major departure from other organisms in terms of nuclear organization. The implications are especially profound with respect to the germline, where it has been widely presumed that the homolog pairing observed during Drosophila meiosis is an extension of the pairing established during embryogenesis [24]–[30]. Notably, there is evidence for homolog pairing being in place during the mitotic divisions immediately preceding meiosis, consistent with it having been established much earlier in development [27], [29], [31]. Indeed, such pre-meiotic pairing has been reported to continue uninterrupted into meiosis [27], [29], [31], which may explain the ability of Drosophila females to maintain interactions associated with meiotic pairing and form the synaptonemal complex (SC) between homologs in the absence of double-strand breaks (DSBs) [32], induction of which is essential for pairing and SC formation in yeast and mammals.

This early pairing in the Drosophila germline is in stark contrast to meiotic pairing in nonDipteran organisms consisting of distinct soma and germline tissues [30], [33]–[35]; while a recent study showed pairing as early as the final round of pre-meiotic replication in mice, there was no demonstration of pairing earlier to this time point [36]. Here we clarify the origin of germline pairing in Drosophila, refuting a long-held hypothesis that it derives from pairing established during embryogenesis and arguing, instead, for a program of germline pairing that is not initiated until the five mitotic cell cycles just prior to meiosis.

## Results and Discussion

### Homologous chromosomes enter the germline unpaired

**Figure 1:**
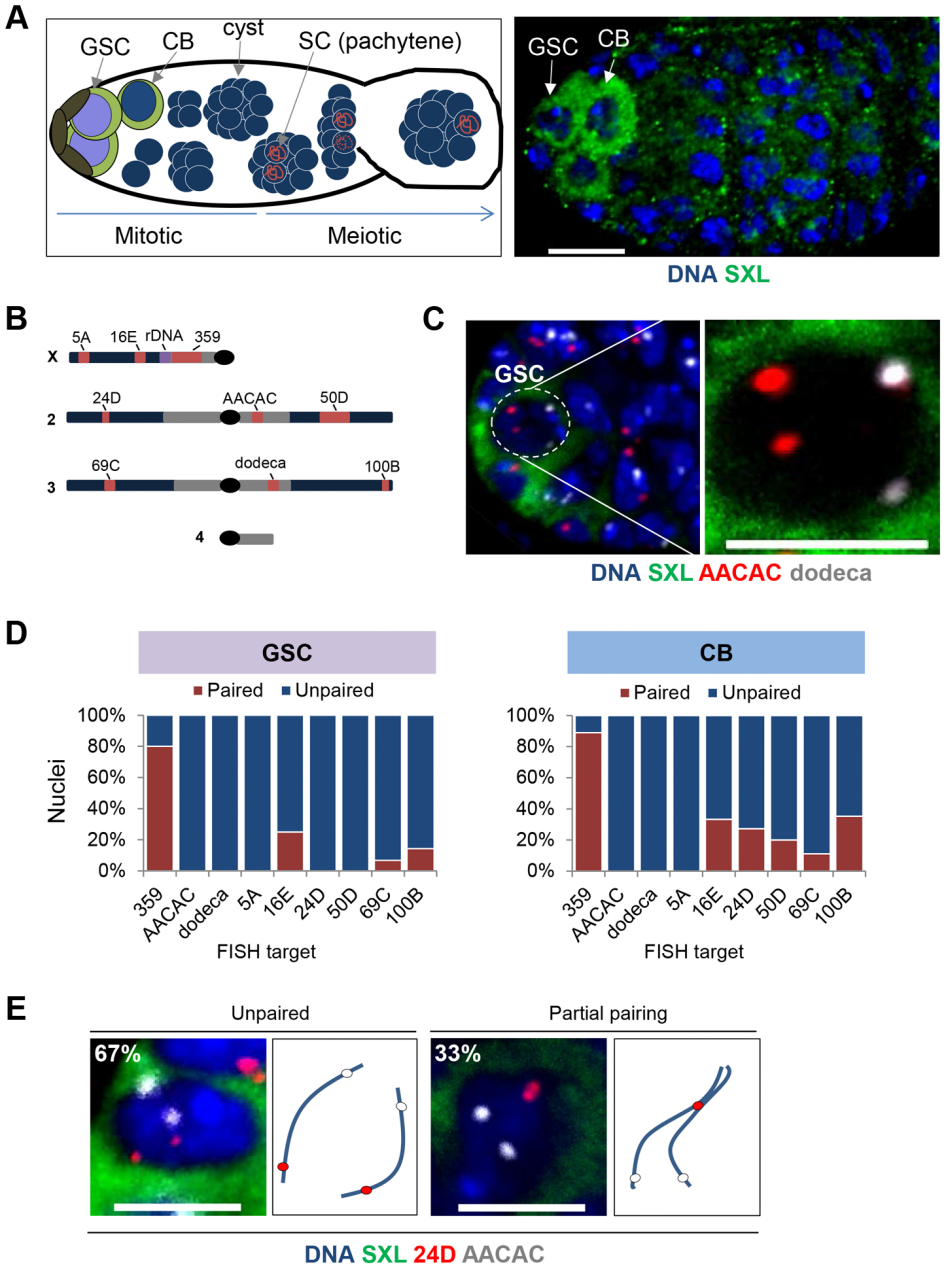
Homologous chromosomes enter the germline unpaired. A, Left: Schematic of a germarium showing pre-meiotic mitotic cell divisions as well as maturation of the meiotic cysts. The GSCs (purple nuclei) are positioned adjacent to the somatic niche (brown) and express high levels of SXL (green cytoplasm). Each GSC divides asymmetrically to produce a renewed stem cell and a differentiating cystoblast (CB, blue nucleus surrounded by green cytoplasm), which is positioned distal to the niche. The CB will undergo four more rounds of mitotic divisions to form a 16-cell cyst. Following these pre-meiotic stages, the 16-cell cyst will enter meiotic prophase, as defined by the initiation (zygotene) and complete formation (pachytene) of the synaptonemal complex (SC, red) between the paired homologs in two of the sixteen cells. Only a single cell will complete meiosis within each 16-cell cyst to form a mature egg (not shown) Arrow, direction of maturation. Right: Wild-type germarium stained for DNA (blue) and SXL (green). A GSC and CB are indicated by arrows and identified by SXL staining and relative position to somatic niche. Approximately 1–2 GSCs and 1–2 CBs are present in each germarium. Scale bar represents 10 µm. B, Drosophila chromosomes and targets of FISH probes (red). Heterochromatin is denoted in grey and rDNA cluster on the X-chromosome is in purple. C, Image of a GSC nucleus (dashed circle) at the tip of a germarium identified by DAPI (blue) surrounded by cytoplasmic SXL (green) staining and combined with FISH targeting AACAC (red) and dodeca (grey). Two signals for each FISH target represent separated homologous loci. Scale bar represents 5 µm. D, Percentage of nuclei exhibiting paired and unpaired loci in GSCs (left panel) and CBs (right panel). 15–30 ovaries were scored for each stage with a combined total of 242 GSC nuclei and 262 CB nuclei (approximately 30 nuclei for each locus at each stage). E, CB nuclei identified with SXL staining in combination with two-color FISH targeting AACAC (grey) and 24D (red) on Chromosome 2. Cartoon depicts hypothetical arrangement of homologous chromosomes as either unpaired or partially paired. Scale bars represents 5 µm.

To determine whether chromosomes are paired prior to meiosis in Drosophila, we first analyzed adult germline stem cells (GSCs), focusing on the female germline, as meiosis in male Drosophila does not follow “the standard meiotic script” [26], [37]. The GSC of the Drosophila female are found in its two ovaries, the very tip of which consists of ∼16–20 germaria, each of which harbors just one or two GSCs positioned adjacent to the somatic niche [38]. During oogenesis, each GSC divides asymmetrically to produce a renewed stem cell and a differentiating cystoblast (CB), which is positioned distal from the niche and destined to undergo four more rounds of replication and division before entering meiotic prophase (Figure 1A). In order to identify the GSCs and distinguish them from subsequent pre-meiotic stages, we took advantage of an antibody to the cytoplasmic protein SXL, levels of which increase in GSCs and then decrease as differentiation proceeds [39].

Chromosome positioning in individual GSC nuclei was assessed by fluorescent in situ hybridization (FISH) in whole-mounted tissues, using techniques that preserve the nuclear architecture followed by high-resolution microscopy and 3D-image reconstruction. Within each nucleus, a single FISH signal or two signals separated by ≤0.8 µm were considered to represent the paired state of the targeted locus. To evaluate the extent of genome-wide pairing, we used nine FISH probes (Figure 1B and Table S1). Three probes targeted highly repeated sequences of the centromeric heterochromatin, including that of the X chromosome (359), chromosome 2 (AACAC), and chromosome 3 (dodeca). The remaining six probes were generated with Oligopaint technology [40] and targeted single copy euchromatic loci, including two loci (5A and 16E) on the X chromosome, loci on the left (24D) and right (50D) arms of chromosome 2 and the left (69C) and right (100B) arms of chromosome 3. Importantly, these probes were extremely efficient, with 100% of nuclei displaying at least one focus for each probe.

In stark contrast to the assumption that meiotic pairing is an extension of pre-existing somatic pairing and that chromosomes therefore enter the germline in the paired state, we observed extensive separation of homologs in GSCs. Eight out of the nine loci produced two distinct FISH signals in the majority of, if not all, nuclei and were considered unpaired in 75–100% of GSC nuclei (Figure 1C–D) in experiments representing 15–30 ovaries. In fact, the average inter-allelic distances for these loci was equivalent to the radius of the nucleus (2.29 µm; p>0.05, unpaired *t* test; Table S1), consistent with a random positioning of the maternal and paternal chromosomes relative to each other. The dramatic deficiency of pairing at these eight loci argues that the genome-wide homolog pairing and subsequent SC formation during meiosis does not derive from a paired state that is extant in the GSC.

The one exception was the X-linked 359 repeat, which was paired in 80% of GSC nuclei (Figure 1D). While this may be indicative of some homolog alignment in GSCs, it may also reflect the proximity of this locus to the rDNA gene cluster, which is spatially confined to the nucleolus, and/or the large size of this repeated region [20], [28], [41], estimated to be 11 Mb in size [42]. Importantly, two other euchromatic loci proximal (16E) and distal (5A) to the 359 repeat were mostly (75%) and completely (100%), respectively, unpaired, indicating that the X-chromosome is not exceptional in its capacity to pair in GSCs. We have also found the 359 repeat to exhibit atypical pairing dynamics in somatic cells [43].

### Homolog pairing is established during the mitotic cell cycles prior to meiosis

The largely unpaired state of GSCs shifted our focus to determining whether, outside of the 359 repeat, pre-meiotic pairing occurs in the female germline to any significant extent. To this end, we looked directly downstream of the GSCs to the differentiating CBs, which number between one and two per germarium and, relative to the GSCs, are positioned downstream of the niche. Here, we targeted the euchromatic loci of 5A, 16E, 24D, 50D, 69C, and 100B (Figure 1D and Table S1) and found unambiguous levels (11–35%) of pairing at all but 5A. These findings establish that Drosophila does, indeed, support at least some degree of pairing well before meiosis initiates.

Despite significant levels of euchromatic pairing in CBs, no pairing was detected at the centromeric repeats AACAC and dodeca, suggesting the partial nature of homolog pairing at this stage. In fact, when we performed two-color FISH targeting two loci across a single arm of chromosome 2 in CB nuclei, we did not detect any pairing of the centromeric locus despite 33% (n = 21) pairing of the chromosome arm (Figure 1E).

**Figure 2:**
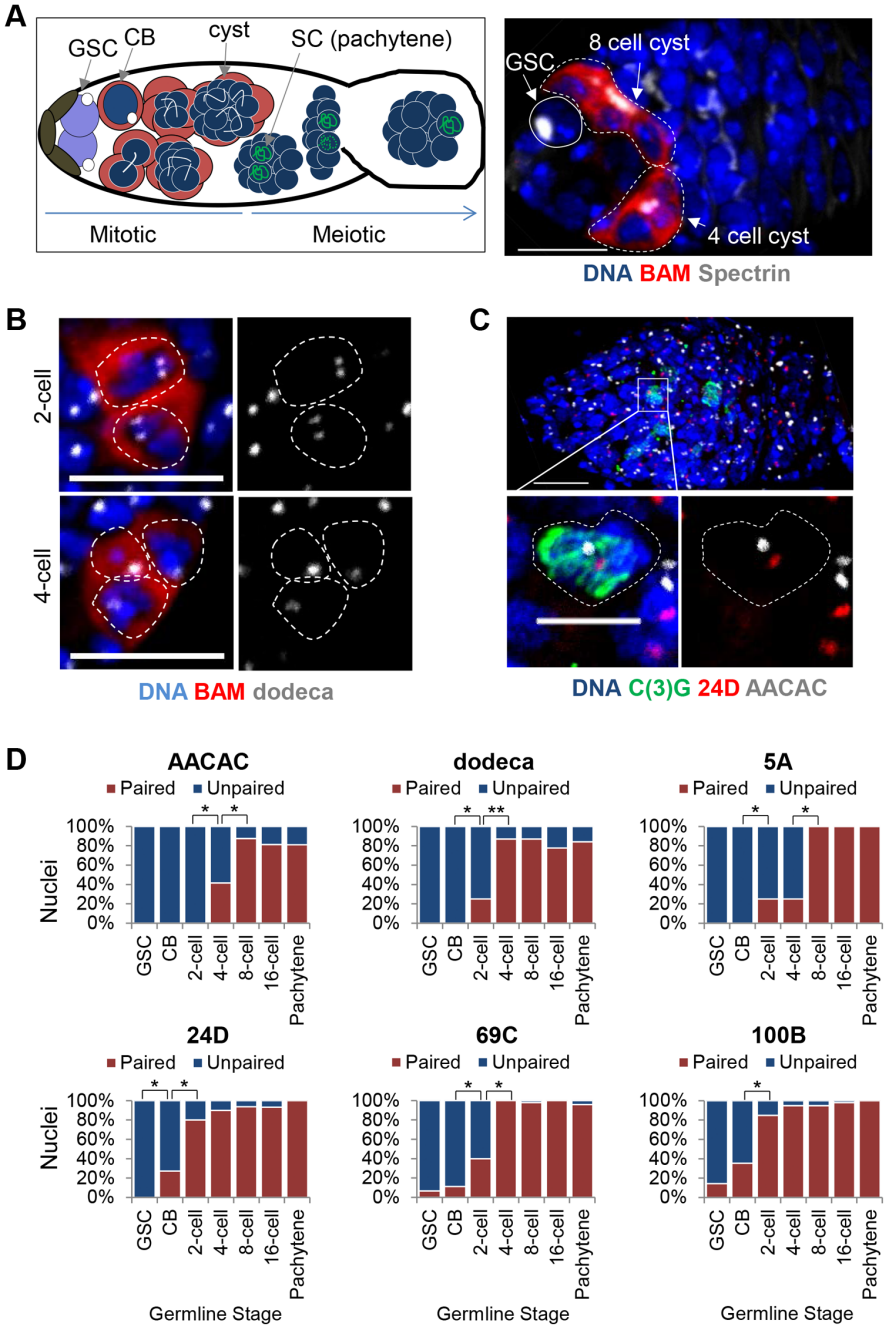
Homolog pairing is established during the mitotic cell cycles prior to meiosis. A, Left: Schematic of a germarium identifying the pre-meiotic stages with BAM (red) and Spectrin (white) and meiotic stages with C(3)G (green). Right: Wild-type germarium in which GSCs are identified by the position near the niche, absence of BAM staining, and presence of a spectrosome (white). Developing cysts are identified by the presence of BAM staining and a branched fusome (white). DAPI, blue. Approximately 1–2 germline cysts are present in each germarium, with equal occurrence of the 2-, 4-, 8-, and 16-cell stages. Scale bar represents 10 µm. B, FISH targeting dodeca (grey). 2-cell and 4-cell cysts (the 4^th^ cell is out of focus) identified with BAM∶GFP (pseudo-colored red). Scale bars represent 10 µm. C, FISH targeting 24D (red) and AACAC (grey) in a germarium identifying pachytene nuclei in meiosis with an antibody against the SC protein C(3)G (green). Scale bar represents 10 µm in upper panel and 5 µm in lower panel. D, Percentage of nuclei exhibiting paired and unpaired loci in pre-meiotic stages as well as in meiotic pachytene with FISH targeting AACAC, dodeca, 5A, 24D, 69C, and 100B. Pre-meiotic cysts were identified using BAM∶GFP and Spectrin. Pachytene nuclei were identified in a separate experiment using an antibody against C(3)G. 15–30 ovaries were scored for each stage with a minimum of 20 nuclei counted for 2- and 4-cell stages, 40 nuclei for the 8-cell stage, and 80 nuclei for the 16-cell stage (*P<0.01, **P<0.0001).

The partial pairing observed in CBs raised the possibility that complete pairing can be achieved in cells progressing through mitotic divisions prior to the initiation of meiosis. In Drosophila, this program includes four rounds of divisions in which the CB becomes a 2-, 4-, 8-, and ultimately 16-cell cyst of interconnected cystocytes, one of which completes meiosis. Importantly, these stages precede the pachytene stage of meiosis, in which homologous chromosomes are fully synapsed (Figure 2A). In order to evaluate the progression of pre-meiotic pairing, we used the *P[bamP-GFP]* transgene, a transcriptional reporter that is not expressed in GSCs or pachytene, but is expressed in each of the intervening pre-meiotic stages [39], [44], along with an antibody against Spectrin, a cytoskeletal protein that forms a spherical structure called the spectrosome in GSCs and CBs, and an antibody against C(3)G, which identifies the SC in meiotic nuclei [45] (Figure 2A–C). Developing cysts were staged based on the number of BAM-positive cells and by Spectrin staining, which, in cysts, localizes to a branched structure called the fusome. We found that homolog pairing levels rapidly increased through the divisions, with each of six FISH targets reaching maximum levels of pairing (87–95%) by the 8-cell cyst (Figure 2D). Once maximum levels of pairing were achieved, they were maintained throughout the remaining pre-meiotic divisions and into the pachytene stage of meiosis (Figure 2C–D), suggesting that homologous chromosomes initiate pairing up to four mitotic divisions prior to meiosis and enter meiosis fully aligned. Importantly, we were able to assess pairing in all four cells of twenty-seven 4-cell cysts, where 87%, 90%, 100%, and 95% of cells showed pairing at dodeca, 24D, 69C, and 100B, respectively. Because the oocyte derives from one cell of a 4-cell cyst, these observations demonstrate that pairing can be observed well before the initiation of meiosis. Analogous data were obtained for the 8-cell and 16-cell cysts.

Interestingly, not all chromosome loci achieved pre-meiotic pairing at the same rate; the 359, 24D, and 100B loci reached maximum levels of pairing prior to the 5A, AACAC, dodeca, and 69C loci by one to four divisions (Figure 1D and Figure 2D). This observation is consistent with the higher level of euchromatic as versus centromeric pairing we observed for autosomes in CBs and suggests that, rather than strictly initiating at the centromeres, where SC formation is first observed [46], [47], germline pairing may initiate at different rates or times across the genome.

### Chromosomes maintain an unpaired state throughout germline development

**Figure 3:**
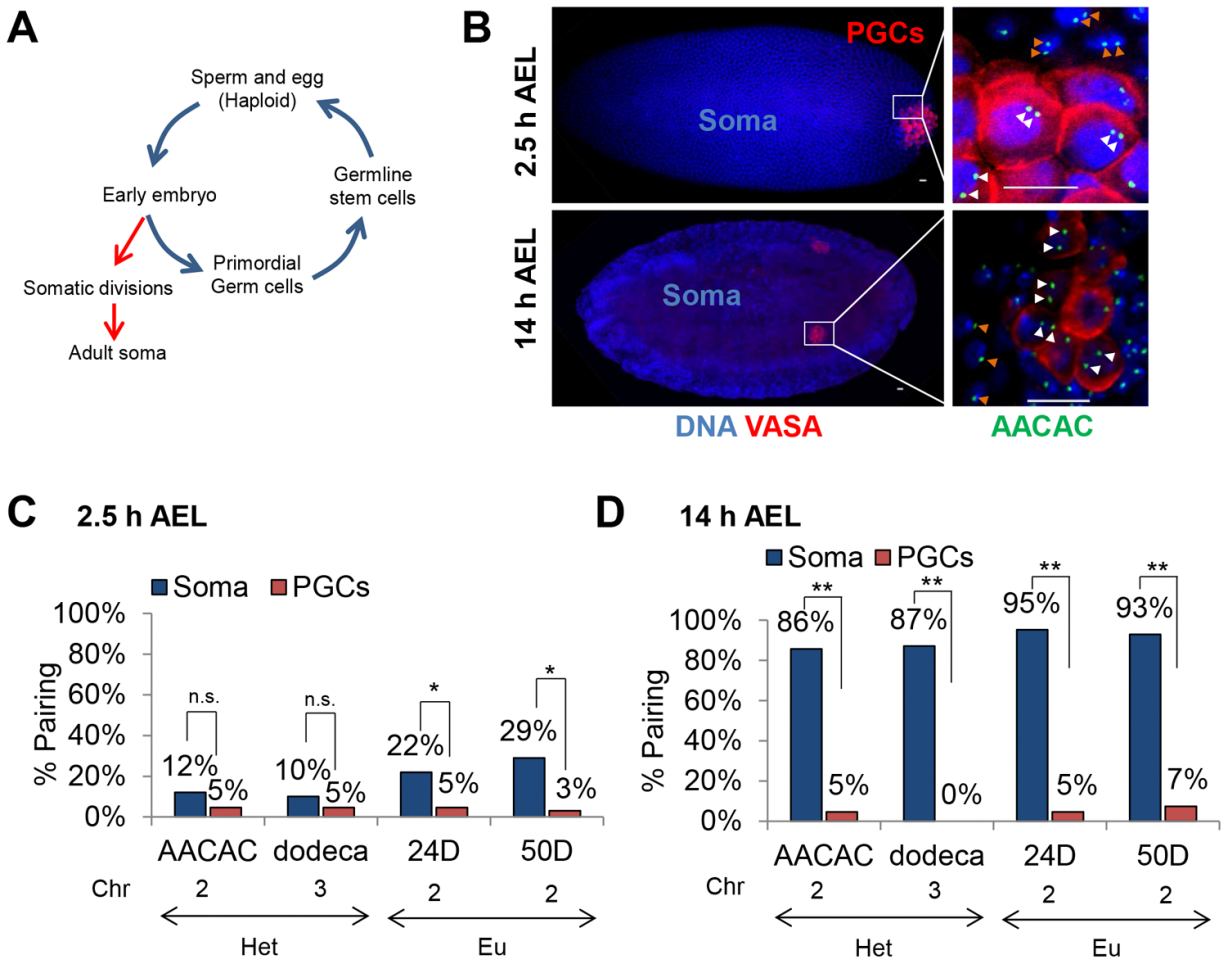
Homolog separation is maintained in primordial germ cells throughout embryogenesis. A, Drosophila life-cycle. The Drosophila embryo develops through a series of synchronized, rapid divisions for the first 2.5 hours (h) after egg lay (AEL). Approximately 8–10 nuclei separate from the somatic divisions, migrate to the posterior pole of the embryo, and, following up to two further divisions, give rise to ∼15–30 primordial germ cells [38]. These cells will eventually produce the adult GSCs, from which haploid gametes are derived. B, Using DAPI (blue) and an antibody to the germline-specific protein VASA (red), primordial germ cells (PGCs) are identified at the posterior pole of embryos 2.5 h AEL and within the embryonic gonad 14 h AEL. Right-most column are magnified images of PGCs and somatic cells at the respective embryonic stages with FISH targeting AACAC (green). White arrowheads denote PGC loci and orange arrowheads denote somatic loci. Scale bars represent 10 µm. C–D, Percentage of pairing in embryos 2.5 h (C) and 14 h (D) AEL within somatic and PGC nuclei (n.s. not significant, *p<0.05, **p<0.0001). The chromatin state (Het, heterochromatin, or Eu, euchromatin), and chromosome are noted below each FISH target. For each data point, 46–98 nuclei were scored from a total of 6–7 embryos (see Materials and Methods).

The results described above refute the long held belief that homolog pairing in Drosophila meiosis is an extension of pairing events established within the embryo and maintained throughout development in all tissues, including the germline. Hence, they encouraged us to assess whether germline cells ever support somatic levels of homolog pairing during development or, alternatively, whether unpairing represents a global nuclear reorganization specifically in the GSCs. We, therefore, evaluated pairing levels during embryogenesis, as this 24-hour phase of development marks both the onset of somatic homolog pairing as well as the separation of germline and somatic lineages. The germline distinguishes itself ∼2 hours after egg lay (AEL), with the primordial germ cells (PGCs), from which adult GSCs are derived, forming at the posterior pole of the embryo and becoming identifiable with the germline-specific protein marker VASA [48] (Figure 3A–B). Examination of homologous pairing during embryogenesis has indicated that some sites attain pairing as early as 2 hours AEL [21]. In fact, the ∼500 Kb histone locus on chromosome 2 has been reported to pair ∼2.5 hours AEL in the soma and PGCs [21], providing reason to believe that PGCs do not differ from somatic cells in their capacity to pair. However, it is unclear if pairing of this locus reflects genome-wide levels or specific features of this locus, such as its transcriptional activity [17], [20], [21], [28], [49], [50]. Here, we distinguish these alternatives by examining the behavior of four other loci across the genome - two centromeric (AACAC and dodeca) and two single-copy euchromatic loci (24D and 50D).

Confirming our ability to detect the onset of pairing in somatic cells, we examined embryos 2.5 hours AEL and observed, respectively, 12% and 10% pairing at the centromeric AACAC and dodeca repeats and 22% and 29% pairing at the euchromatic 24D and 50D loci (Figure 3C). In contrast, the PGCs at the posterior pole of the embryos consistently exhibited lower levels of pairing at this stage for all four loci, ranging from 3% to 5% (Figure 3C). We next analyzed embryonic nuclei 14 hours AEL and found 80–100% of somatic nuclei were paired for each of the four loci (Figure 3D), levels consistent with full attainment of somatic homolog pairing [21]. Strikingly, however, homologous chromosomes in the PGCs, which at 14 hours AEL are in contact with somatic cells, remained essentially unpaired, attaining only 0–7% pairing at any of the four loci (p<0.0001; Figure 3D). In these cells, the inter-allelic distances were extensive, averaging 2.5–3.5 µm and, in some cases, reaching as much as 5–6 µm (Figure S1). Additionally, sex-specific differences were not observed, suggesting that, regardless of sex, germline progenitors do not support genome-wide homolog pairing (Figure S2). Thus, PGCs maintain a predominantly unpaired state of homologous chromosomes throughout embryogenesis. These observations argue that germ cells are never exposed to the widespread pairing observed in somatic cells and thus, represent the only Drosophila tissue identified so far that escapes this phenomenon.

### Germline progenitors have large nuclear volumes with chromosomes juxtaposed to the nuclear envelope

**Figure 4:**
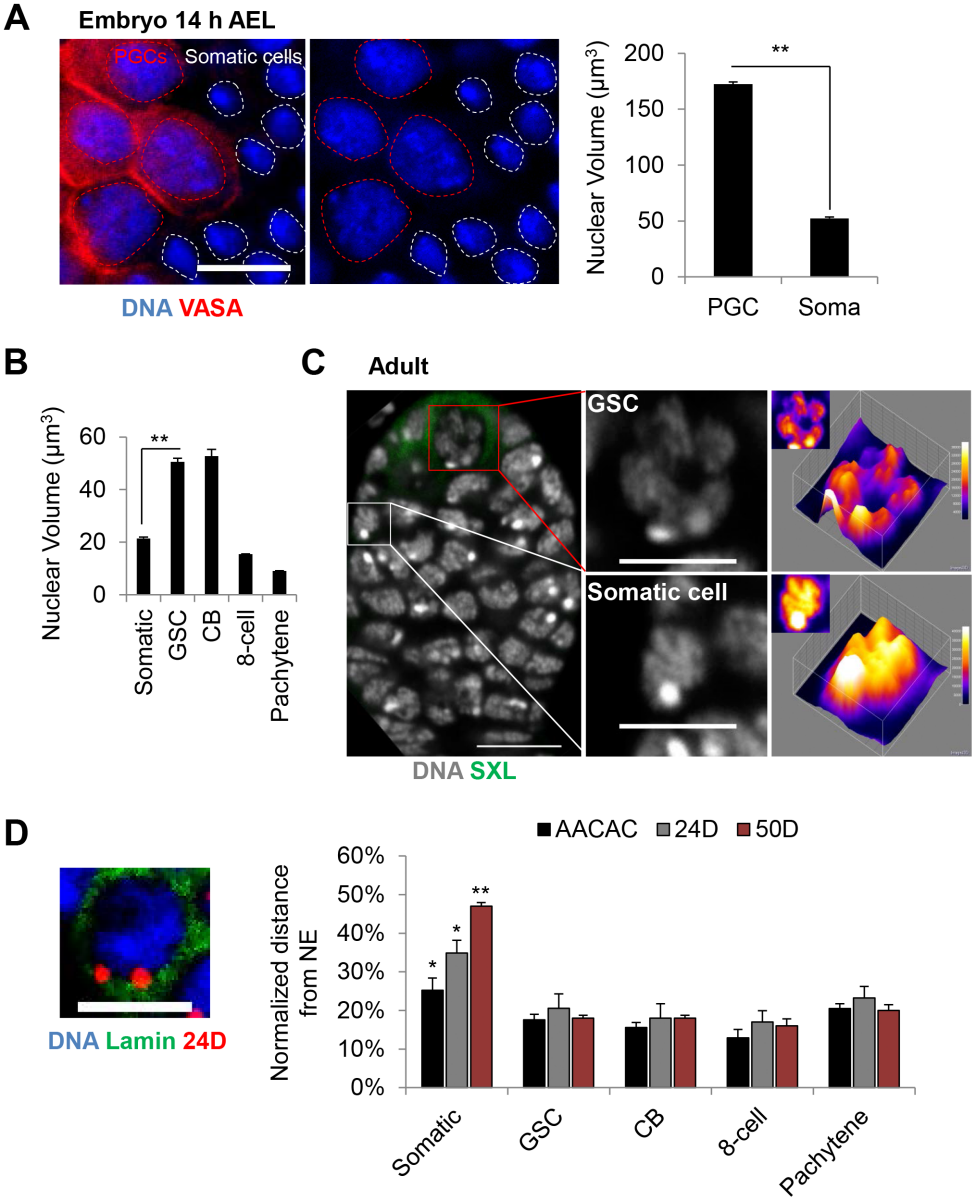
Germline progenitors contain large nuclear volumes with chromosomes juxtaposed to the nuclear envelope. A, PGC nuclei (VASA, red) are larger than surrounding somatic nuclei in embryonic gonads 14 h AEL. DAPI, blue. Dashed circles denote nuclear periphery. Scale bar represents 10 µm. Right: Average nuclear volume of PGCs and surrounding somatic cells ± SEM. B, Average nuclear volume of germline and somatic follicle cells of the adult ovary ± SEM (**p<0.0001). C, Wild-type germarium stained for DNA (grey) and SXL (green). Shown on right are cross-sections of representative GSC and somatic nuclei with 3D and 2D (insets) surface plots displaying increased peripheral intensity in the nucleus of the GSC and more uniform intensity in the nucleus of the somatic cell. Scale bar represents 10 µm in the image of the germarium and 5 µm in images of single nuclei. D, Left: GSC nucleus with FISH targeting 24D (red) and lamin staining (nuclear envelope, green). Scale bar represents 5 µm. Right: Average distance between FISH signals and the nuclear envelope (NE) ± SEM, normalized to the nuclear radius, in germline and somatic follicle cells of the adult ovary. Asterisks denote significant differences in the normalized distances between somatic and GSCs (*p<0.05, **p<0.0001). For each data point, a minimum number of 30 nuclei were scored (see Materials and Methods).

To better understand how germline progenitors maintain an unpaired state, we determined whether they might exhibit other distinctive features of nuclear organization. Notably, we observed that the nuclear volumes of PGCs 14 hours AEL (172.5 µm^3^) were ∼3.3 fold greater than that of neighboring somatic cells (52.3 µm^3^, p<0.0001; Figure 4A) and reasoned that larger nuclear volumes could cause increased distances between homologs and thus account for the lower levels of pairing in PGCs. Consistent with this hypothesis, the nuclear volumes of GSCs and CBs (50.5–52.7 µm^3^) were greater than two times larger than that of the surrounding somatic follicle cells (21.3 µm^3^, p = 0.0027; Figure 4B), while those of the 8-cell cysts and cells in pachytene were approximately the same or smaller in size (Figure 4B). To test the potential of larger nuclear volumes to explain lower levels of pairing, we normalized inter-allelic distances to the nuclear radius. This analysis revealed that, even when inter-allelic distances were normalized, the level of pairing in PGCs remained less than that observed in somatic cells by six to nineteen fold (Figure S3). This outcome suggests that the separation of homologous chromosomes cannot be fully explained by nuclear volume alone.

We next analyzed the global distribution of DNA within the larger GSC nuclei and found a distinct nuclear 4′,6-diamidino-2-phenylindole (DAPI) staining pattern compared to somatic follicle cells, indicating a change in chromatin structure. As shown in Figure 4C, surface plots of DAPI fluorescence intensity revealed a non-uniform peripheral staining pattern in GSC nuclei. In contrast, the DAPI fluorescence intensity in adjacent somatic follicle cell nuclei typically displayed a relatively uniform and diffuse staining pattern (Figure 4C). To assess whether this DNA distribution could result from the juxtaposition of chromosomes to the nuclear envelope, we used FISH in combination with an antibody against lamin to measure the distance between the nuclear envelope and each of three loci across chromosome 2: the AACAC centromeric repeat and the 24D and 50D loci in the middle of each arm. As predicted, all three loci were equally close to the nuclear envelope in GSCs, with average distances of only 15–20% of the total nuclear radius (Figure 4D). Similar results were found for the nuclei of CB, 8-cell cysts, pachytene cells, as well as embryonic PGCs (Figure 4D and Figure S4), indicating that the peripheral localization of chromosomes is adopted early in germline development and maintained into meiosis. In contrast, the same three loci in somatic cells exhibited greater distances from the nuclear envelope, averaging of 25–47% of the radius, with the centromeric locus closest to the nuclear envelope (Figure 4D and Figure S4). This arrangement, in which centromeres are located in the periphery with chromosome arms displaced across the nuclear space, is consistent with the Rabl configuration of chromosomes frequently found in Drosophila somatic cells [51], [52]. We conclude that germline cells may adopt a distinct nuclear structure which, compared to somatic cells, involves placement of chromosomes in close proximity to the nuclear envelope along their entire length.

### Conclusion

Our findings reveal extensive separation of homologous chromosomes in germline progenitors from early embryogenesis until the five mitotic cell cycles just prior to meiosis and, in this regard, align Drosophila with other organisms that establish homolog pairing *de novo* in the gonad. Importantly, our observations are in agreement with Christophorou *et al.* (also in this issue), who also found a deficiency of homolog pairing in the GSCs of the Drosophila adult female. The lack of pairing in germline progenitors is especially noteworthy, considering the widespread prevalence of pairing in the somatic tissues of Drosophila. Why should an organism ensure homologous chromosomes remain unpaired in germline progenitors, only to allow pairing beyond the stem cell divisions? One possibility is that, since germline progenitors generate the entire cell population responsible for transmitting the genome to subsequent generations, any negative outcome of that pairing could be propagated to a much greater extent as compared to undesired events occurring downstream of the GSCs and thus have a higher probability of multigenerational consequences.

**Figure 5:**
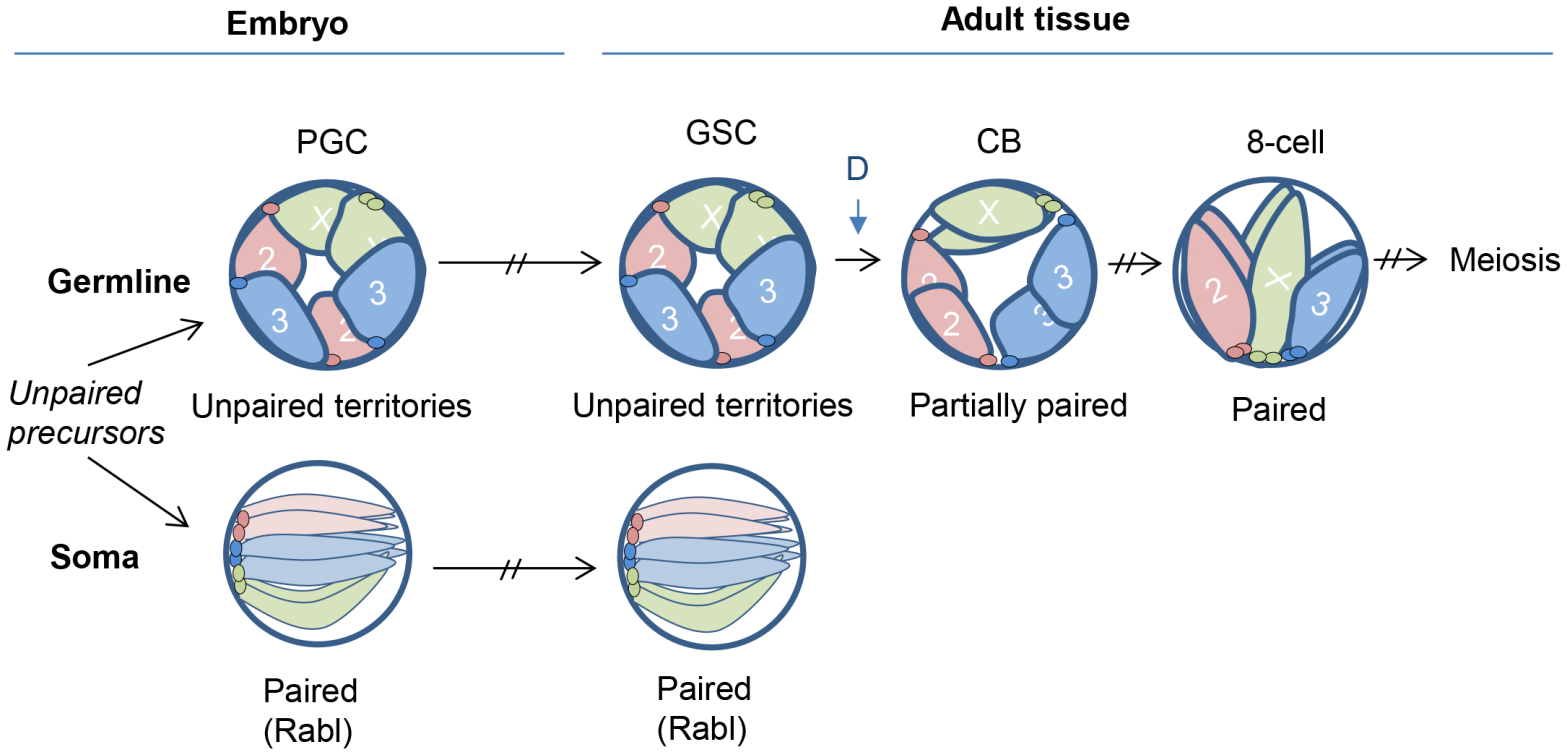
Model for germline nuclear organization. Once a germline cell fate is established in early embryogenesis, homologous chromosomes remain unpaired and localize to the nuclear periphery, creating non-overlapping chromosome territories that may block ectopic pairing. This organization is maintained through development and into the adult GSCs. Germline pairing initiates coincident with germline differentiation (time point denoted as ‘D’ during the pre-meiotic mitotic divisions, ultimately leading to complete homolog alignment and the initiation of meiosis and SC formation. In somatic cells, homologous chromosomes instead adopt a configuration that permits, or even promotes, pairing. Such a configuration might be the Rabl organization, which occurs in early embryogenesis and positions centromeres and telomeres at opposite nuclear poles [21].

Our discovery of unpaired homologs in germline progenitors also demonstrates that homolog pairing is not an inevitable feature of Drosophila chromosomes and is consistent with studies arguing that pairing is a controlled process reflecting genes that promote pairing as well as those that antagonize it [14], [15], [22], [23], [43], [53]. Here, we further propose that potentially undesirable homologous interactions are precluded in Drosophila germline progenitors coordinately with, or due to, the separation of the progenitors from the soma in early embryogenesis. Pairing could also be precluded through a localization of chromosomes to the nuclear periphery. Such a configuration could lead to the formation of chromosome territories that separate homologs in the germline, as opposed to configurations that permit or even promote the pairing observed in the soma (Figure 5). Note that our data do not clarify whether the mechanisms that pair homologous chromosomes in somatic cells are distinct from, or similar to, those that eventually pair homologous chromosomes in the pre-meiotic cells. Nevertheless, to the extent that the mechanisms may be different, our findings are consistent with the notion that germline nuclei may suppress or delay the mechanisms that support pairing in the soma, perhaps through nuclear organization, while, in the pre-meiotic cells, simultaneously permit a separate mechanism that promotes pairing. Indeed, Christophorou *et al.* (also in this issue) show that pre-meiotic pairing is perturbed in the absence of meiosis-specific proteins such as components of the SC, suggesting that the mechanisms of pre-meiotic pairing cannot be entirely similar to that of somatic pairing.

Interestingly, we found that chromosomes maintain their peripheral localization even during the pre-meiotic 8-cell stage when homologs are fully aligned and continue to maintain this configuration into meiosis. Whether this localization is a significant aspect of pre-meiotic pairing will be of interest, as chromosome interactions with the nuclear envelope have been proposed to promote meiotic pairing in several organisms [54]–[57], as well as influence polytene pairing in Drosophila [58]. Regardless, our observations establish a distinction between the organization of paired chromosomes in pre-meiotic nuclei (peripheral localization) and that in somatic nuclei (internal localization) in Drosophila.

In closing, we return to the extraordinary degree of pairing that Drosophila and other Dipteran insects support in their soma. If homologous interactions can lead to negative outcomes, why do these organisms permit a near-organismal wide level of such interactions? One explanation is that somatic homolog interactions may, under some circumstances, confer advantages [12], [28], [34], [59]–[63] and, consistent with this, transient and localized instances of somatic homolog interactions have been documented or at least implicated in a wide variety of organisms, including mammals [1]–[7], [12], [63]–[65]. Indeed, in light of our discovery that the different tissues of Drosophila can have dramatically different levels of pairing it is possible that greater scrutiny of nonDipteran species will reveal many more instances of somatic pairing and, hence, evidence that somatic pairing is a widespread potential of genomes in general [12], [14], [43].

## Materials and Methods

### Drosophila strains

Drosophila stocks and crosses were maintained on a standard medium at 25°C. For wild-type, we used the *y^1^ w^1118^* strain. To identify the BAM protein that was used to distinguish the pre-meiotic cyst stages, we crossed *y^1^ w^1118^* to a strain carrying the transgene *P(bamP-GFP)* [44], a kind gift from Michael Buszcak (The University of Texas Southwestern Medical Center at Dallas).

### Generation of FISH probes

Oligo probes for the 359, AACAC, and dodeca heterochromatic repeats [42], [66] were synthesized with either a 5′ Cy5 or Tye3 fluorescent dye by Integrated DNA Technologies (IDT). The design of the probe sequences were previously described [43] and are as follows: 359: GGGATCGTTAGCACTGGTAATTAGCTGC, AACAC: AACACAACACAACACAACACAACACAACACAACAC, and dodeca: ACGGGACCAGTACGG. Oligo probes were resuspended in 1×TE at 100 µM concentration and stored at −20°C.

Euchromatic probes 5A (4E2-5C10), 16E (16B3-17A2), 24D (24D1-24F1), 50D (50D1-53C7), 69C (69A1-69E6), and 100B (100B9-100D1) were designed and generated using the Oligopaint technology [40]. Briefly, a library of 7500 (24D and 100B), 10000 (5A, 16E, and 69C), and 25000 (50D) unique oligos (MYcroarray) were designed for amplification. Each library was amplified using a common 5′ Cy3-conjugated forward primer (5′-CGCTCGGTCTCCGTTCGTCTC) and unlabeled reverse primer (5′-GGGCTAGGTACAGGGTTCAGCgcaatg).

### Antibodies

The antibodies and dilutions used were mouse anti-SXL (m18, Developmental Studies Hybridoma Bank [DSHB], 1∶10), rat anti-VASA (DSHB, 1∶300), mouse anti-Spectrin (DSHB 3A9, 1∶50), mouse anti-lamin (DSHB ADL84.12, 1∶100) and rabbit anti-GFP (Molecular Probes, 1∶300). The mouse anti-C(3)G (1A8-1G2, 1∶200) antibody was a gift from Scott Hawley (Stowers Institute, Kansas City, Missouri).

The secondary antibodies were DyLight 488 goat anti-rabbit (Jackson Labs) used at 1∶165, Cy3 labeled goat anti-rat (Jackson Labs) used at 1∶100 and DyLight 488 goat anti-mouse (Jackson Labs) used at 1∶100.

### Immunofluorescence and FISH of Drosophila ovaries

Immunostaining was performed prior to FISH, using a modified protocol of [45]. Females (∼10–15 per experiment) were aged 3–4 days in the presence of males and were fed yeast paste overnight prior to dissection. Ovaries were isolated in PBS and immediately fixed for 10 min in 200 µl of PBS containing 4% formaldehyde and 0.5% Nonidet P-40, plus 600 µl Heptane. Fixed ovaries were then rinsed three times in PBT (PBS plus 0.2% Tween-20), and washed three times for 5 min in PBT. Prior to immunostaining, the ovaries were teased apart and blocked by incubating in PBT plus 1.5% BSA at room temperature for 1 hour. Primary antibodies were incubated at 4°C overnight in PBT. Three 20-min washes in PBT were performed prior to incubation with secondary antibodies at room temperature for 2 hours, followed by two 20-min washes in PBT and one 10-min wash in PBS.

For FISH, PBS buffer was replaced with 2×SSCT (0.3 M NaCl, 0.03 M NaCitrate, 0.1% Tween-20) by three quick washes. After washing, the ovaries were then gradually exchanged into 2×SSCT/50% formamide with 10-min washes in 2×SSCT/20% formamide, then in 2×SSCT/40% formamide, and then two washes in 2×SSCT/50% formamide. Ovaries were then allowed to settle and the 2×SSCT/50% formamide was removed prior to the addition of 36 µl of hybridization solution (2×SSCT, 50% formamide, 10% (w/v) dextran sulfate, RNase) and up to 4 µl of probe. For heterochromatic targets, 100 pmol of probe was added to the hybridization. For single-copy euchromatic targets, 200–400 pmol of Oligopaint probes were added to the hybridization. To preserve the nuclear structure, chromosomes were denatured at 78°C in a thermal cycler for 30 min followed by incubation overnight at 37°C in the dark. Following hybridization, we performed two 30-min washes of 2×SSCT/50% formamide at 37°C, followed by a 10-min wash in 2×SSCT/20% formamide at room temperature and three quick washes in 2×SSCT. After settling, excess 2×SSCT was removed and the ovaries were mounted in Slowfade mounting media with DAPI (Invitrogen).

### Collection and fixation of Drosophila embryos

We collected 2.5 hour- and 14 hour-old embryos for 1 hour on apple juice plates and then aged them an additional 1.5 or 13 hours, respectively. 2.5 hours after egg lay (AEL) should capture embryos in the final 10 min of cell cycle 13 and the first 50 min of cell cycle 14. Due to the time spent manipulating embryos during the dechorionation step (see below), most embryos were aged ∼5–10 min longer before development was stopped during fixation. During imaging, embryos were also staged by the number and position of primordial germ cells, which are separated from the soma at the pole 2.5 hours AEL and encapsulated within the embryonic gonad 14 hours AEL (Figure 3B).

After collection, we dechorionated the embryos by submerging them in 50% bleach for 90 seconds, followed by a thorough wash in ddH_2_O. For fixation, embryos were placed in PBS containing 4% formaldehyde, 0.5% Nonidet P-40, and 50 mM EGTA, plus 500 µl Heptane for 30 min. The aqueous phase was removed and replaced with 500 µl MeOH and mixed vigorously for 2 min. The embryos were allowed to settle and were washed two times in 100% MeOH and stored for up to a week at −20°C. Prior to immunostaining, the embryos were rehydrated in PBT. Immunostaining and FISH were then performed as described above for ovaries.

### Microscopy and image analysis

All images were collected using a Zeiss LSM780 laser scanning confocal microscope with a 63×, N.A. 1.40 lens. We imaged whole germaria by collecting 200 nm optical sections through the entire tissue at 1024×1024 or 512×512 resolutions with a digital zoom of 3.0. The analysis of the images was performed by both 3D-image reconstruction and examining one section at a time using the Zeiss ZEN 2011 software. FISH foci were counted manually within each nucleus and the distance between the centers of allelic signals was measured using the Ortho – distance function, which permits length measurements in 3D space. 100% of nuclei examined in this study exhibited at least a single FISH signal, indicating high hybridization efficiency. Therefore, a single signal was considered two foci with an inter-signal distance of 0 µm. In some cases where noted, we normalized the inter-signal distances by the radius of the nucleus. In these cases, p values were determined by an unpaired *t* test.

Two homologs were considered paired if the distance between their focus centers was ≤0.8 µm or FISH produced a single signal. To determine the significance between paired states, p values were calculated by a two-tailed Fisher's exact test.

To image the pre-meiotic 2-, 4-, 8-, and 16-cell cysts, we focused on that region of the germarium identified by *P[bamP-GFP]* expression, set the upper and lower limits of the scanned region to capture the entirety of the cysts, and then scored those nuclei that were fully contained within the scanned region and were also unambiguously distinguished from other cell types. This strategy enabled us to score 93–100% of the cells in any chosen cyst.

To image germline cells in the embryo, we focused on that region identified by VASA expression, set the upper and lower limits of the scanned region to capture the majority of the germline cells, and then scored only those nuclei that were fully contained within the scanned region and were also unambiguously distinguished from somatic cells. This strategy enabled us to score the majority of germline cells in any chosen embryo. The somatic embryonic cells that were scored were those that were within the scanned region containing the scored germline cells.

### Measuring nuclear volume

Nuclear envelopes were labeled with an anti-lamin antibody. Nuclear volumes were calculated based on the nuclear diameter using the equation V = 4/3πr^3^.

### Measuring distance between nuclear envelope and FISH signals

Nuclear envelopes were labeled with an anti-lamin antibody. The ZEN software package was then used to measure the shortest distance between FISH signals and the nuclear envelope in 3D space. When two FISH signals were present in the nucleus, only the shortest distance of the two was scored. p values were determined by an unpaired *t* test.

## Supporting Information

Figure S1 Extensive separation of homologs in primordial germ cells. Relative frequencies of inter-allelic distances in embryonic PGCs and somatic cells 14 hours AEL based on FISH targeting AACAC (upper-left), dodeca (upper-right), 24D (lower-left), and 50D (lower-right). The percentage of nuclei exhibiting paired loci for this data set is presented in Fig. 3.
(TIF)

Figure S2 Male and female germline progenitors do not support genome-wide homolog pairing. A, Primordial germ cells (PGCs, VASA, red) in female embryos 14 hours AEL were distinguished from male embryos based on their expression of the female-specific cytoplasmic protein SXL (green). B, Percentage of nuclei paired at AACAC (left-most graph) and 24D (right-most graph) in male and female PGCs as compared to somatic cells in embryos 14 hours AEL. No significant difference in pairing levels were observed between the sexes. For each data point, a minimum number of 20 PGCs and 50 somatic nuclei were scored from a total of 6–7 independent embryos (see Materials and Methods).
(TIF)

Figure S3 Homolog separation in primordial germ cells is not dependent on nuclear size. Distances between allelic signals by FISH (Figure S1) were normalized to the radius of the nucleus to account for the larger nuclear volumes in PGCs as compared to that in somatic cells in embryos 14 h AEL. Despite this normalization, there was 6- (AACAC), 7- (dodeca), 16- (24D), and 19- (50D) times less pairing in PGCs than in somatic cells (**p<0.0001).
(TIF)

Figure S4 Chromosomes from primordial germ cells are in close proximity to the nuclear envelope. A, PGC nucleus with FISH targeting 24D (red, arrowheads) and lamin staining (nuclear envelope, green). Scale bar represents 5 µm. B, Average distance between 24D FISH signals and the nuclear envelope (NE) normalized to the nuclear radius ± SEM in embryonic PGCs as compared to somatic cells 14 hours AEL.
(TIF)

Table S1 Homolog pairing frequencies in GSCs and CBs.
(DOCX)

## References

[1] Marahrens Y (1999) X-inactivation by chromosomal pairing events. Genes Dev 13: 2624–2632. 10541548 10.1101/gad.13.20.2624

[2] Bacher CP, Guggiari M, Brors B, Augui S, Clerc P, et al (2006) Transient colocalization of X-inactivation centres accompanies the initiation of X inactivation. Nat Cell Biol 8: 293–299. 16434960 10.1038/ncb1365

[3] Xu N, Tsai CL, Lee JT (2006) Transient homologous chromosome pairing marks the onset of X inactivation. Science 311: 1149–1152. 16424298 10.1126/science.1122984

[4] Brandt VL, Hewitt SL, Skok JA (2010) It takes two: Communication between homologous alleles preserves genomic stability during V(D)J recombination. Nucleus 1: 23–29. 21327101 10.4161/nucl.1.1.10595 PMC3035125

[5] LaSalle JM, Lalande M (1996) Homologous association of oppositely imprinted chromosomal domains. Science 272: 725–728. 8614834 10.1126/science.272.5262.725

[6] Riesselmann L, Haaf T (1999) Preferential S-phase pairing of the imprinted region on distal mouse chromosome 7. Cytogenet Cell Genet 86: 39–42. 10516430 10.1159/000015426

[7] Gandhi M, Evdokimova VN, K TC, Nikiforova MN, Kelly LM, et al (2012) Homologous chromosomes make contact at the sites of double-strand breaks in genes in somatic G0/G1-phase human cells. Proc Natl Acad Sci U S A 109: 9454–9459. 22645362 10.1073/pnas.1205759109 PMC3386068

[8] Croft JA, Bridger JM, Boyle S, Perry P, Teague P, et al (1999) Differences in the localization and morphology of chromosomes in the human nucleus. J Cell Biol 145: 1119–1131. 10366586 10.1083/jcb.145.6.1119 PMC2133153

[9] Cremer M, von Hase J, Volm T, Brero A, Kreth G, et al (2001) Non-random radial higher-order chromatin arrangements in nuclei of diploid human cells. Chromosome Res 9: 541–567. 11721953 10.1023/a:1012495201697

[10] Bolzer A, Kreth G, Solovei I, Koehler D, Saracoglu K, et al (2005) Three-dimensional maps of all chromosomes in human male fibroblast nuclei and prometaphase rosettes. PLoS Biol 3: e157. 15839726 10.1371/journal.pbio.0030157 PMC1084335

[11] Cremer T, Cremer M (2010) Chromosome territories. Cold Spring Harbor perspectives in biology 2: a003889. 20300217 10.1101/cshperspect.a003889 PMC2829961

[12] Apte MS, Meller VH (2012) Homologue pairing in flies and mammals: gene regulation when two are involved. Genet Res Int 2012: 430587. 22567388 10.1155/2012/430587 PMC3335585

[13] Cavalli G, Misteli T (2013) Functional implications of genome topology. Nature structural & molecular biology 20: 290–299. 10.1038/nsmb.2474 PMC6320674 23463314

[14] Williams BR, Bateman JR, Novikov ND, Wu CT (2007) Disruption of topoisomerase II perturbs pairing in drosophila cell culture. Genetics 177: 31–46. 17890361 10.1534/genetics.107.076356 PMC2013714

[15] Hartl TA, Smith HF, Bosco G (2008) Chromosome alignment and transvection are antagonized by condensin II. Science 322: 1384–1387. 19039137 10.1126/science.1164216

[16] Coulthard AB, Nolan N, Bell JB, Hilliker AJ (2005) Transvection at the vestigial locus of Drosophila melanogaster. Genetics 170: 1711–1721. 15944352 10.1534/genetics.105.041400 PMC1449749

[17] Ou SA, Chang E, Lee S, So K, Wu CT, et al (2009) Effects of chromosomal rearrangements on transvection at the yellow gene of Drosophila melanogaster. Genetics 183: 483–496. 19667134 10.1534/genetics.109.106559 PMC2766311

[18] Stevens N (1908) A study of the germ cells of certain Diptera, with reference to the heterochromosomes and phenomena of synapsis. J Exp Zool 5: 359–374.

[19] Metz CW (1916) Chromosome studies on the Diptera. II. The paired association of chromosomes in the Diptera and its significance. J Exptl Zool 21: 213–279.

[20] Hiraoka Y, Dernburg AF, Parmelee SJ, Rykowski MC, Agard DA, et al (1993) The onset of homologous chromosome pairing during Drosophila melanogaster embryogenesis. J Cell Biol 120: 591–600. 8425892 10.1083/jcb.120.3.591 PMC2119536

[21] Fung JC, Marshall WF, Dernburg A, Agard DA, Sedat JW (1998) Homologous chromosome pairing in Drosophila melanogaster proceeds through multiple independent initiations. J Cell Biol 141: 5–20. 9531544 10.1083/jcb.141.1.5 PMC2132734

[22] Fritsch C, Ploeger G, Arndt-Jovin DJ (2006) Drosophila under the lens: imaging from chromosomes to whole embryos. Chromosome Res 14: 451–464. 16821139 10.1007/s10577-006-1068-z

[23] Bateman JR, Wu CT (2008) A genomewide survey argues that every zygotic gene product is dispensable for the initiation of somatic homolog pairing in Drosophila. Genetics 180: 1329–1342. 18791221 10.1534/genetics.108.094862 PMC2581938

[24] Roeder GS (1997) Meiotic chromosomes: it takes two to tango. Genes Dev 11: 2600–2621. 9334324 10.1101/gad.11.20.2600

[25] Weiner BM, Kleckner N (1994) Chromosome pairing via multiple interstitial interactions before and during meiosis in yeast. Cell 77: 977–991. 8020104 10.1016/0092-8674(94)90438-3

[26] Vazquez J, Belmont AS, Sedat JW (2002) The dynamics of homologous chromosome pairing during male Drosophila meiosis. Curr Biol 12: 1473–1483. 12225662 10.1016/s0960-9822(02)01090-4

[27] Sherizen D, Jang JK, Bhagat R, Kato N, McKim KS (2005) Meiotic recombination in Drosophila females depends on chromosome continuity between genetically defined boundaries. Genetics 169: 767–781. 15545646 10.1534/genetics.104.035824 PMC1449117

[28] McKee BD (2004) Homologous pairing and chromosome dynamics in meiosis and mitosis. Biochim Biophys Acta 1677: 165–180. 15020057 10.1016/j.bbaexp.2003.11.017

[29] Gong WJ, McKim KS, Hawley RS (2005) All paired up with no place to go: pairing, synapsis, and DSB formation in a balancer heterozygote. PLoS Genet 1: e67. 16299588 10.1371/journal.pgen.0010067 PMC1285065

[30] Joyce EF, McKim KS (2007) When specialized sites are important for synapsis and the distribution of crossovers. Bioessays 29: 217–226. 17295219 10.1002/bies.20531

[31] Grell RF, Day JW (1970) Chromosome pairing in the oogonial cells of Drosophila melanogaster. Chromosoma 31: 434–445. 5490309 10.1007/BF00285834

[32] McKim KS, Green-Marroquin BL, Sekelsky JJ, Chin G, Steinberg C, et al (1998) Meiotic synapsis in the absence of recombination. Science 279: 876–878. 9452390 10.1126/science.279.5352.876

[33] Gerton JL, Hawley RS (2005) Homologous chromosome interactions in meiosis: diversity amidst conservation. Nat Rev Genet 6: 477–487. 15931171 10.1038/nrg1614

[34] Zickler D (2006) From early homologue recognition to synaptonemal complex formation. Chromosoma 115: 158–174. 16570189 10.1007/s00412-006-0048-6

[35] Bhalla N, Dernburg AF (2008) Prelude to a division. Annual review of cell and developmental biology 24: 397–424. 10.1146/annurev.cellbio.23.090506.123245 PMC4435778 18597662

[36] Boateng KA, Bellani MA, Gregoretti IV, Pratto F, Camerini-Otero RD (2013) Homologous Pairing Preceding SPO11-Mediated Double-Strand Breaks in Mice. Dev Cell 24: 196–205. 23318132 10.1016/j.devcel.2012.12.002 PMC3562373

[37] McKee BD, Yan R, Tsai JH (2012) Meiosis in male Drosophila. Spermatogenesis 2: 167–184. 23087836 10.4161/spmg.21800 PMC3469440

[38] Williamson A, Lehmann R (1996) Germ cell development in Drosophila. Annual review of cell and developmental biology 12: 365–391. 10.1146/annurev.cellbio.12.1.365 8970731

[39] Chau J, Kulnane LS, Salz HK (2009) Sex-lethal facilitates the transition from germline stem cell to committed daughter cell in the Drosophila ovary. Genetics 182: 121–132. 19237687 10.1534/genetics.109.100693 PMC2674811

[40] Beliveau BJ, Joyce EF, Apostolopoulos N, Yilmaz F, Fonseka CY, et al (2012) Versatile design and synthesis platform for visualizing genomes with Oligopaint FISH probes. Proc Natl Acad Sci U S A 109: 21301–21306. 23236188 10.1073/pnas.1213818110 PMC3535588

[41] Tsai JH, McKee BD (2011) Homologous pairing and the role of pairing centers in meiosis. Journal of cell science 124: 1955–1963. 21625006 10.1242/jcs.006387

[42] Dernburg AF (2011) In situ hybridization to somatic chromosomes in Drosophila. Cold Spring Harbor protocols 2011 10.1101/pdb.top065540 21880819

[43] Joyce EF, Williams BR, Xie T, Wu CT (2012) Identification of genes that promote or antagonize somatic homolog pairing using a high-throughput FISH-based screen. PLoS Genet 8: e1002667. 22589731 10.1371/journal.pgen.1002667 PMC3349724

[44] Chen D, McKearin D (2003) Dpp signaling silences bam transcription directly to establish asymmetric divisions of germline stem cells. Curr Biol 13: 1786–1791. 14561403 10.1016/j.cub.2003.09.033

[45] Page SL, Hawley RS (2001) c(3)G encodes a Drosophila synaptonemal complex protein. Genes Dev 15: 3130–3143. 11731477 10.1101/gad.935001 PMC312841

[46] Takeo S, Lake CM, Morais-de-Sa E, Sunkel CE, Hawley RS (2011) Synaptonemal complex-dependent centromeric clustering and the initiation of synapsis in Drosophila oocytes. Curr Biol 21: 1845–1851. 22036182 10.1016/j.cub.2011.09.044

[47] Tanneti NS, Landy K, Joyce EF, McKim KS (2011) A pathway for synapsis initiation during zygotene in Drosophila oocytes. Curr Biol 21: 1852–1857. 22036181 10.1016/j.cub.2011.10.005 PMC12823172

[48] Santos AC, Lehmann R (2004) Germ cell specification and migration in Drosophila and beyond. Curr Biol 14: R578–589. 15268881 10.1016/j.cub.2004.07.018

[49] Xu M, Cook PR (2008) The role of specialized transcription factories in chromosome pairing. Biochim Biophys Acta 1783: 2155–2160. 18706455 10.1016/j.bbamcr.2008.07.013

[50] Lee AM, Wu CT (2006) Enhancer-promoter communication at the yellow gene of Drosophila melanogaster: diverse promoters participate in and regulate trans interactions. Genetics 174: 1867–1880. 17057235 10.1534/genetics.106.064121 PMC1698615

[51] Rabl C (1985) Uber Zelltheilung. Morphol Jahrb 10: 214–330.

[52] Marshall WF, Dernburg AF, Harmon B, Agard DA, Sedat JW (1996) Specific interactions of chromatin with the nuclear envelope: positional determination within the nucleus in Drosophila melanogaster. Molecular biology of the cell 7: 825–842. 8744953 10.1091/mbc.7.5.825 PMC275932

[53] Bateman JR, Larschan E, D'Souza R, Marshall LS, Dempsey KE, et al (2012) A genome-wide screen identifies genes that affect somatic homolog pairing in Drosophila. G3 (Bethesda) 2: 731–740. 22870396 10.1534/g3.112.002840 PMC3385979

[54] Phillips CM, Meng X, Zhang L, Chretien JH, Urnov FD, et al (2009) Identification of chromosome sequence motifs that mediate meiotic pairing and synapsis in C. elegans. Nat Cell Biol 11: 934–942. 19620970 10.1038/ncb1904 PMC4001799

[55] Penkner A, Tang L, Novatchkova M, Ladurner M, Fridkin A, et al (2007) The nuclear envelope protein Matefin/SUN-1 is required for homologous pairing in C. elegans meiosis. Dev Cell 12: 873–885. 17543861 10.1016/j.devcel.2007.05.004

[56] Sato A, Isaac B, Phillips CM, Rillo R, Carlton PM, et al (2009) Cytoskeletal forces span the nuclear envelope to coordinate meiotic chromosome pairing and synapsis. Cell 139: 907–919. 19913287 10.1016/j.cell.2009.10.039 PMC2825574

[57] Lee CY, Conrad MN, Dresser ME (2012) Meiotic chromosome pairing is promoted by telomere-led chromosome movements independent of bouquet formation. PLoS Genet 8: e1002730. 22654677 10.1371/journal.pgen.1002730 PMC3359977

[58] Bauer CR, Hartl TA, Bosco G (2012) Condensin II promotes the formation of chromosome territories by inducing axial compaction of polyploid interphase chromosomes. PLoS Genet 8: e1002873. 22956908 10.1371/journal.pgen.1002873 PMC3431300

[59] Wu CT, Morris JR (1999) Transvection and other homology effects. Curr Opin Genet Dev 9: 237–246. 10322135 10.1016/S0959-437X(99)80035-5

[60] Duncan IW (2002) Transvection effects in Drosophila. Annu Rev Genet 36: 521–556. 12429702 10.1146/annurev.genet.36.060402.100441

[61] Kassis JA (2002) Pairing-sensitive silencing, polycomb group response elements, and transposon homing in Drosophila. Adv Genet 46: 421–438. 11931233 10.1016/s0065-2660(02)46015-4

[62] Kennison JA, Southworth JW (2002) Transvection in Drosophila. Adv Genet 46: 399–420. 11931232 10.1016/s0065-2660(02)46014-2

[63] Grant-Downton RT, Dickinson HG (2004) Plants, pairing and phenotypes–two's company? Trends in genetics : TIG 20: 188–195. 15041173 10.1016/j.tig.2004.02.005

[64] Barzel A, Kupiec M (2008) Finding a match: how do homologous sequences get together for recombination? Nat Rev Genet 9: 27–37. 18040271 10.1038/nrg2224

[65] Galagan JE, Selker EU (2004) RIP: the evolutionary cost of genome defense. Trends Genet 20: 417–423. 15313550 10.1016/j.tig.2004.07.007

[66] Dernburg AF, Sedat JW (1998) Mapping three-dimensional chromosome architecture in situ. Methods in cell biology 53: 187–233. 9348510 10.1016/s0091-679x(08)60880-8

