# Supplementary figures and images for: Germline Progenitors Escape the Widespread Phenomenon of Homolog Pairing during Drosophila Development
Source: PLoS Genet. 2013 Dec 19;9(12):e1004013. doi: 10.1371/journal.pgen.1004013 (PMC3868550; doi:10.1371/journal.pgen.1004013)

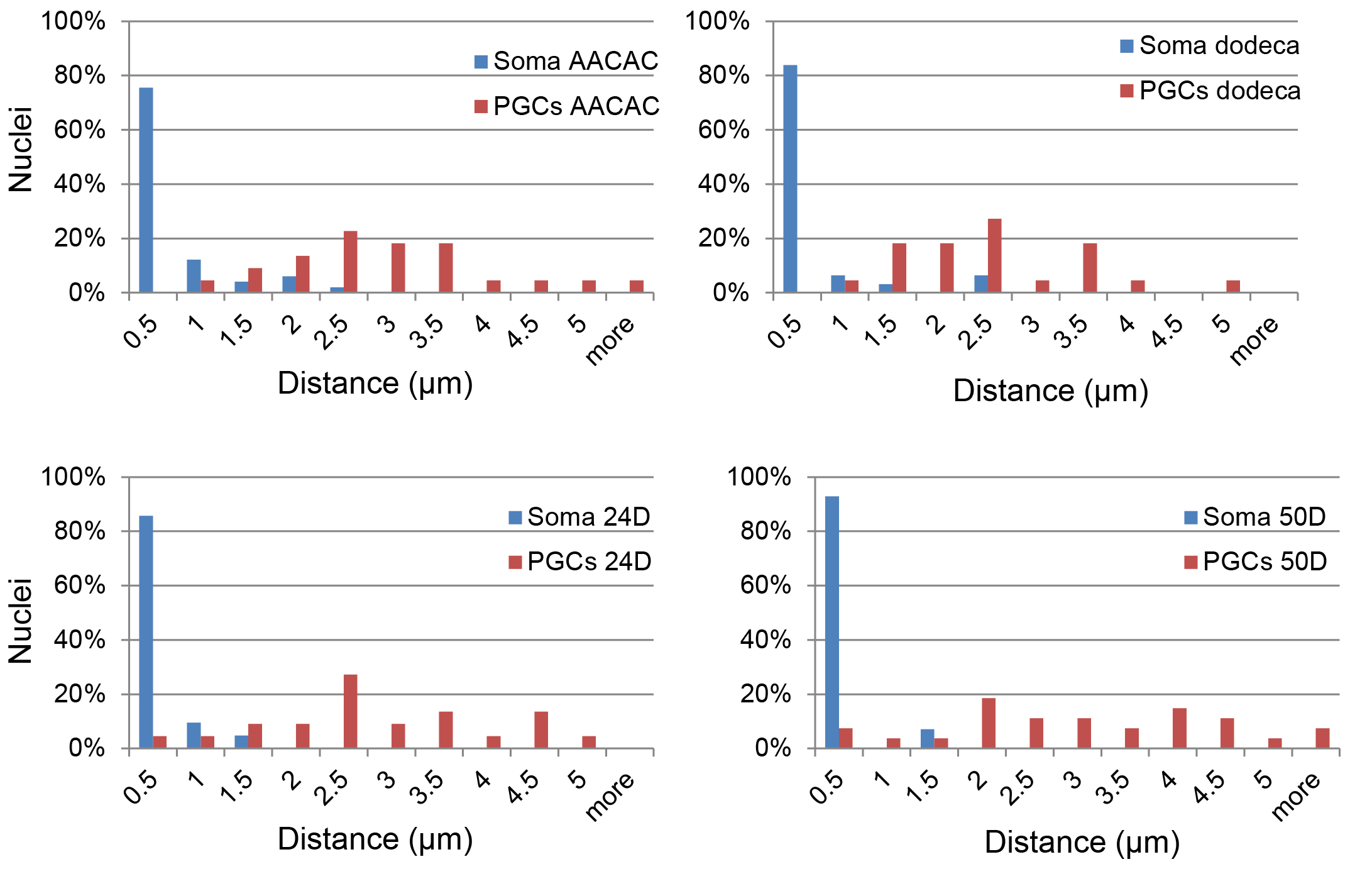

Supplement: Figure S1 — Extensive separation of homologs in primordial germ cells. Relative frequencies of inter-allelic distances in embryonic PGCs and somatic cells 14 hours AEL based on FISH targeting AACAC (upper-left), dodeca (upper-right), 24D (lower-left), and 50D (lower-right). The percentage of nuclei exhibiting paired loci for this data set is presented in Fig. 3. (TIF) [file pgen.1004013.s001.tif]

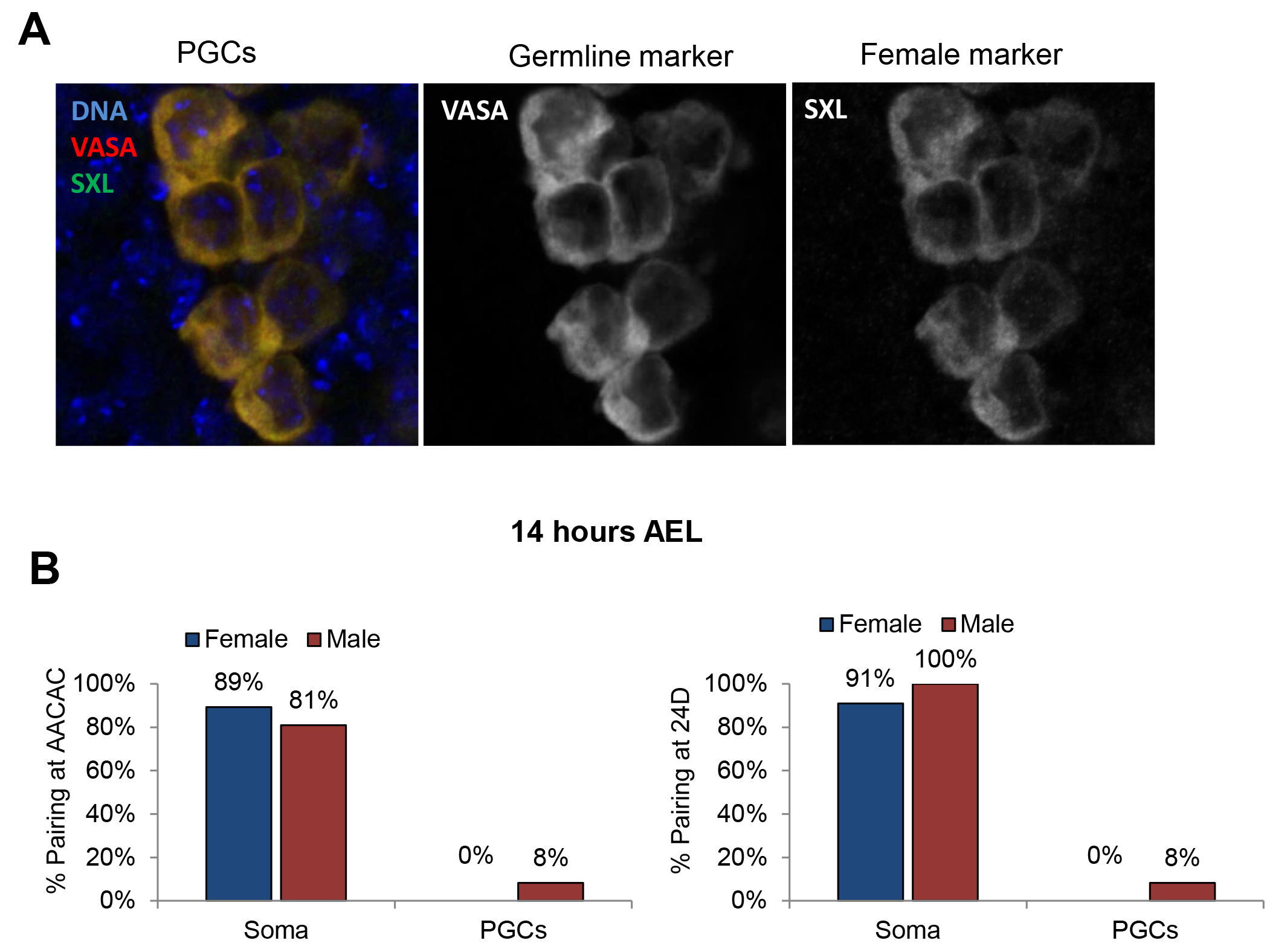

Supplement: Figure S2 — Male and female germline progenitors do not support genome-wide homolog pairing. A, Primordial germ cells (PGCs, VASA, red) in female embryos 14 hours AEL were distinguished from male embryos based on their expression of the female-specific cytoplasmic protein SXL (green). B, Percentage of nuclei paired at AACAC (left-most graph) and 24D (right-most graph) in male and female PGCs as compared to somatic cells in embryos 14 hours AEL. No significant difference in pairing levels were observed between the sexes. For each data point, a minimum number of 20 PGCs and 50 somatic nuclei were scored from a total of 6–7 independent embryos (see Materials and Methods). (TIF) [file pgen.1004013.s002.tif]

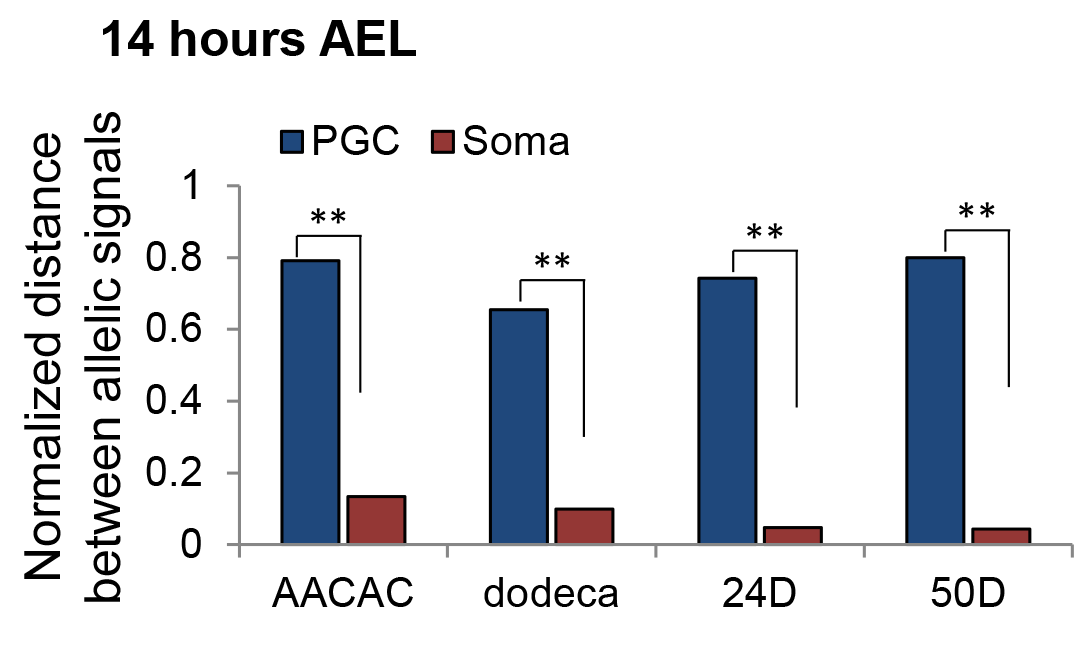

Supplement: Figure S3 — Homolog separation in primordial germ cells is not dependent on nuclear size. Distances between allelic signals by FISH (Figure S1) were normalized to the radius of the nucleus to account for the larger nuclear volumes in PGCs as compared to that in somatic cells in embryos 14 h AEL. Despite this normalization, there was 6- (AACAC), 7- (dodeca), 16- (24D), and 19- (50D) times less pairing in PGCs than in somatic cells (**p<0.0001). (TIF) [file pgen.1004013.s003.tif]

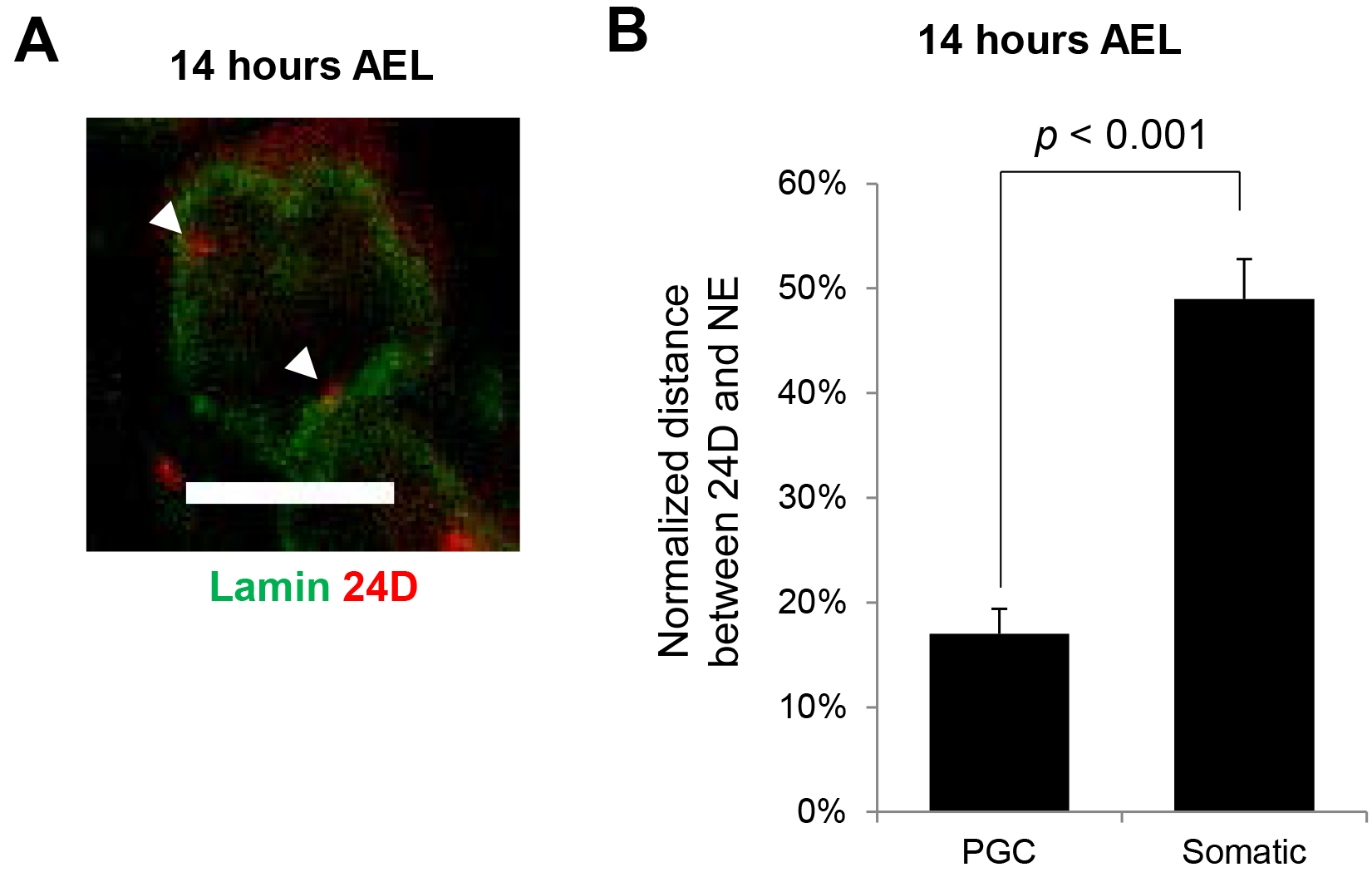

Supplement: Figure S4 — Chromosomes from primordial germ cells are in close proximity to the nuclear envelope. A, PGC nucleus with FISH targeting 24D (red, arrowheads) and lamin staining (nuclear envelope, green). Scale bar represents 5 µm. B, Average distance between 24D FISH signals and the nuclear envelope (NE) normalized to the nuclear radius ± SEM in embryonic PGCs as compared to somatic cells 14 hours AEL. (TIF) [file pgen.1004013.s004.tif]
