# Supplementary material for: Germline Progenitors Escape the Widespread Phenomenon of Homolog Pairing during Drosophila Development
Source: PLoS Genet. 2013 Dec 19;9(12):e1004013. doi: 10.1371/journal.pgen.1004013 (PMC3868550; doi:10.1371/journal.pgen.1004013)
Supplement: Table S1 — Homolog pairing frequencies in GSCs and CBs. (DOCX) [file pgen.1004013.s005.docx]

**Table S1. Homolog pairing frequencies in GSCs and CBs.**

Joyce *et al.*2013

| Cell type | FISH probe | Target | Target size | Single-signal^1^ | Pairing^2^ | Avg. distance^3^ | n^4^ |
| --- | --- | --- | --- | --- | --- | --- | --- |
| GSC | 359 | X | Het^5^ | 87.5% | 87.5% | 0.20 (0.6) | 16 |
|  | AACAC | 2R | Het^5^ | 0.0% | 0.0% | 2.36 (0.7) | 48 |
|  | dodeca | 3R | Het^5^ | 0.0% | 0.0% | 2.59 (0.6) | 16 |
|  | 5A | X | 672 Kb | 0.0% | 0.0% | 2.28 (0.2) | 19 |
|  | 16E | X | 700 Kb | 25.0% | 25.0% | 1.38 (0.8) | 28 |
|  | 24D | 2L | 491Kb | 0.0% | 0.0% | 2.57 (1.0) | 27 |
|  | 50D | 2R | 2.7Mb | 0.0% | 0.0% | 2.54 (0.9) | 22 |
|  | 69C | 3L | 674 Kb | 6.7% | 6.7% | 2.55 (1.2) | 30 |
|  | 100B | 3R | 462Kb | 0.0% | 0.0% | 2.55 (0.9) | 36 |
| CB | 359 | X | Het^5^ | 75.0% | 83.3% | 0.27 (0.4) | 24 |
|  | AACAC | 2R | Het^5^ | 0.0% | 0.0% | 2.50 (1.1) | 20 |
|  | dodeca | 3R | Het^5^ | 0.0% | 0.0% | 2.00 (0.6) | 48 |
|  | 5A | X | 672 Kb | 0.0% | 0.0% | 1.48 (0.48) | 24 |
|  | 16E | X | 700 Kb | 33.3% | 33.3% | 1.03 (0.5) | 30 |
|  | 24D | 2L | 491Kb | 18.2% | 27.3% | 1.13 (0.7) | 44 |
|  | 69C | 3L | 674 Kb | 11.1% | 11.1% | 1.14 (0.3) | 18 |
|  | 50D | 2R | 2.7Mb | 10.0% | 20.0% | 1.42 (0.7) | 20 |
|  | 100B | 3R | 462Kb | 29.4% | 35.3% | 1.09 (0.8) | 34 |

^1^Percentage of germline stem cell (GSC) or cystoblast (CB) nuclei with a single FISH signal.

^2^Percentage of nuclei with a single FISH signal or two signals <0.8 µm apart.

^3^Average distance between FISH signals. A single signal was scored as a distance of 0 µm. Standard deviation are noted in parentheses.

^4^n = total number of nuclei scored

^5^Het = heterochromatin. Exact target sizes are unknown but 359 is estimated to be ~11 Mb.
